# Human periprostatic adipose tissue promotes prostate cancer aggressiveness *in vitro*

**DOI:** 10.1186/1756-9966-31-32

**Published:** 2012-04-02

**Authors:** Ricardo Ribeiro, Cátia Monteiro, Virgínia Cunha, Maria José Oliveira, Mariana Freitas, Avelino Fraga, Paulo Príncipe, Carlos Lobato, Francisco Lobo, António Morais, Vítor Silva, José Sanches-Magalhães, Jorge Oliveira, Francisco Pina, Anabela Mota-Pinto, Carlos Lopes, Rui Medeiros

**Affiliations:** 1 Molecular Oncology Group-CI, Portuguese Institute of Oncology, Porto, Portugal; 2 Abel Salazar Biomedical Sciences Institute, University of Porto, Porto, Portugal; 3 Research Department-Portuguese League Against Cancer (NRNorte), LPCC, Porto, Portugal; 4 Biomaterials Division, NEWTherapies Group, INEB, Porto, Portugal; 5 Department of Pathology and Oncology, Faculty of Medicine, Porto, Portugal; 6 General Pathology Laboratory, Faculty of Medicine, University of Coimbra, Coimbra, Portugal; 7 CIMAGO, Centre of Investigation in Environment, Genetics and Oncobiology, Faculty of Medicine, University of Coimbra, Coimbra, Portugal; 8 CNC, Centre of Neurosciences and Cell Biology, University of Coimbra, Coimbra, Portugal; 9 Urology Department, Porto Hospital Centre, Porto, Portugal; 10 Urology Department, Porto Military Hospital, Porto, Portugal; 11 Urology Department, Portuguese Institute of Oncology, Porto, Portugal; 12 Urology Department, S. João Hospital, Porto, Portugal; 13 Molecular Oncology Group - CI, Portuguese Institute of Oncology, Porto Centre, Edifício Laboratórios - Piso 4, Rua Dr. António Bernardino Almeida, 4200-072 Porto, Portugal

**Keywords:** Adipose tissue, Cell line, Cell proliferation, Cell tracking, Obesity, Periprostatic, Prostate cancer

## Abstract

**Background:**

Obesity is associated with prostate cancer aggressiveness and mortality. The contribution of periprostatic adipose tissue, which is often infiltrated by malignant cells, to cancer progression is largely unknown. Thus, this study aimed to determine if periprostatic adipose tissue is linked with aggressive tumor biology in prostate cancer.

**Methods:**

Supernatants of whole adipose tissue (explants) or stromal vascular fraction (SVF) from paired fat samples of periprostatic (PP) and pre-peritoneal visceral (VIS) anatomic origin from different donors were prepared and analyzed for matrix metalloproteinases (MMPs) 2 and 9 activity. The effects of those conditioned media (CM) on growth and migration of hormone-refractory (PC-3) and hormone-sensitive (LNCaP) prostate cancer cells were measured.

**Results:**

We show here that PP adipose tissue of overweight men has higher MMP9 activity in comparison with normal subjects. The observed increased activities of both MMP2 and MMP9 in PP whole adipose tissue explants, likely reveal the contribution of adipocytes plus stromal-vascular fraction (SVF) as opposed to SVF alone. MMP2 activity was higher for PP when compared to VIS adipose tissue. When PC-3 cells were stimulated with CM from PP adipose tissue explants, increased proliferative and migratory capacities were observed, but not in the presence of SVF. Conversely, when LNCaP cells were stimulated with PP explants CM, we found enhanced motility despite the inhibition of proliferation, whereas CM derived from SVF increased both cell proliferation and motility. Explants culture and using adipose tissue of PP origin are most effective in promoting proliferation and migration of PC-3 cells, as respectively compared with SVF culture and using adipose tissue of VIS origin. In LNCaP cells, while explants CM cause increased migration compared to SVF, the use of PP adipose tissue to generate CM result in the increase of both cellular proliferation and migration.

**Conclusions:**

Our findings suggest that the PP depot has the potential to modulate extra-prostatic tumor cells' microenvironment through increased MMPs activity and to promote prostate cancer cell survival and migration. Adipocyte-derived factors likely have a relevant proliferative and motile role.

## Background

In recent years substantial evidence has been provided for the linkage between adipose tissue dysfunction and cancer progression [1,2]. Excess accumulation of adipose tissue corresponds by definition to obesity, which has been associated with prostate cancer aggressiveness [3,4].

In prostate cancer, the extra-capsular extension of cancer cells into the periprostatic (PP) fat is a pathological factor related with worst prognosis [5]. It is now well established that the interactions between non-tumor cells in the microenvironment and the tumor cells are decisive of whether cancer cells progress towards metastasis or whether they remain dormant [6].

Prostate cancer cells generated within prostatic acini frequently infiltrate and even surpass the prostatic capsule, therefore interacting with the surrounding PP adipose tissue. Previous work showed that such adipose tissue has the potential to modulate prostate cancer aggressiveness, through the increased production of adipokines, namely interleukin 6 (IL-6) [7]. Moreover, a recent report showed an association of PP adipose tissue thickness with prostate cancer severity [8].

Different studies have demonstrated the critical influence of adipose tissue-derived factors in cancer cells [9-11], including prostate tumor cells [12-14]. Together, these reports indicate that factors produced by adipose tissue, particularly adipocytes may stimulate the progression of cancer cells. However, to our knowledge, the influence of PP adipose tissue-derived factors on prostate cancer cells has not been exploited. Noteworthy, we previously observed that prostate cancer induced the increase of PP adipose metabolic activity, promoting a favorable environment for aggressive tumor biology [15].

To address these issues, we first studied the gelatinolytic profile of PP whole adipose tissue and its respective stromal-vascular fraction. Next, we used PP adipose tissue-derived conditioned medium to analyze *in vitro *its influence in proliferation and migration of prostate cancer cells.

## Methods

### Patients and collection of human PP adipose tissue

Men diagnosed with clinically localized prostate cancer or nodular prostatic hyperplasia (BPH) and eligible for retropubic radical prostatectomy or prostate surgery of nodular hyperplasia, without other major co-morbidities, were included in this study after informed consent agreement. The project was approved by the ethics committees of the participating Hospitals. Human anterior-lateral PP and pre-peritoneal visceral (VIS) samples of adipose tissue were collected during surgery and immediately processed.

### Adipose tissue primary cultures and preparation conditioned media (CM)

PP and VIS adipose tissue fragments were processed to primary whole adipose tissue (explants) cultures using a modified protocol from Thalmann et al. [16]. Briefly, after incubation of explants (0.3 g/mL) for 16 hours in DMEM/F12 (Gibco) medium, supplemented with biotin 16 μM (Sigma Aldrich), panthotenate 18 μM (Sigma Aldrich), ascorbate 100 μM (Sigma Aldrich), and 1% penicillin-streptomycin (Sigma Aldrich) (sDMEM/F12), fresh medium was added, and was referred to as time zero for time-course experiments. Explant cultures were maintained at 37°C and 5% CO_2_. After 48 hours, the undernatant was collected, centrifuged (20 000 g,3 minutes), aliquoted and stored at -80°C as explant conditioned medium (CM).

Other pieces of VIS and PP adipose tissue were incubated with collagenase (2 mg/mL) (Collagenase A, Roche) for 60 minutes at 37°C with agitation (120 rpm). After removal of adipocytes layer, the supernatant was discarded and the stromal-vascular fraction (SVF) cell pellet resuspended in sDMEM/F-12 with 10% Newborn Calf Serum (NCS) (Sigma Aldrich) and filtered through a 40 μm cell strainer (BD Falcon, BD Biosciences). Following erythrocyte lysis (Buffer EL, QIAgen), SVFs were resuspended and seeded (500 μL of cell suspension) in wells coated with 0.2% gelatin (Sigma Aldrich) in sDMEM/F-12 medium with 10% NCS. Stromal-vascular fraction cells were maintained at 37°C and 5% CO_2_. After 48 hours, fresh medium free from NCS was added. Forty-eight hours after this time-point CM was collected, centrifuged at 20 000 g for 3 minutes and the supernatant stored at -80°C as SVF CM.

### Human PC-3 and LNCaP cell lines

PC-3 and LNCaP cell lines were obtained from the European Collection of Cell Cultures (ECCAC) and from the American Type Cell Culture (ATCC), respectively. Both cell lines were maintained in RPMI 1640 medium, supplemented with (%) L-glutamine and (%) Hepes (Gibco), 10% FBS (Gibco) and 1% PS (Sigma Aldrich), at 37°C with 5% CO_2_.

### Cell proliferation

Cancer cells were seeded into 96-well plates (5×10^3 ^and 10×10^3 ^cells/well for PC-3 and LNCaP cells, respectively) and incubated for 24 hours in RPMI 1640 medium with 10% FBS. Next, supernatant was removed and new cell medium free from FBS, with (50% volume) or without (control) adipose tissue-derived conditioned medium was added to cancer cells.

Media was removed after 24 hours, and cells were stored at -80°C. Then, the pellet was solubilized in a lysis buffer supplemented with a DNA-binding dye (CyQUANT cell proliferation assay, Invitrogen). DNA content was evaluated in each well by fluorimetry at 480/535 nm using a standard curve previously generated for each cell type, after plotting measured fluorescence values in samples *vs *cell number, as determined from cell suspensions using a hemocytometer. Samples were performed in duplicate and the mean value used for analyses.

### Zymography

Gelatinolytic activities of MMP2 and MMP9 of supernatants from adipose tissue primary cultures were determined on substrate impregnated gels. Briefly, total protein from supernatants of primary cultures of adipose tissue (12 μg/well), were separated on 10% SDS-PAGE gels containing 0.1% gelatin (Sigma-Aldrich). After electrophoresis a 30 minutes washing step (2% Triton X-100) was performed, and gels were incubated 16-18 h at 37°C in substrate buffer (50 mM Tris-HCl, pH7.5, 10 mM CaCl_2_), to allow MMP reactivation. Next, gels were stained in a solution with Comassie Brilliant Blue R-250 (Sigma-Aldrich), 40% methanol and 10% acetic acid for 30 minutes. The correspondent MMP2 and MMP9 clear lysed bands were identified based on their molecular weight and measured with a densitometer (Quantity One, BioRad).

### Cell tracking and analysis of cellular motility

For the time-lapse microscopy analysis (Zeiss Axiovert inverted-fluorescence microscope), exponentially growing cancer cells were seeded into 96-well plates at a density of 5×10^3 ^and 10×10^3 ^cells/well, for PC-3 and LNCaP, respectively. After 24 hours incubation in RPMI 1640 media supplemented with 10% FBS, supernatant was removed and new medium with (50% volume) or without (control, 0% CM) adipose tissue-derived conditioned medium, were added to cancer cells. At this time point the time-lapse experiment was started. A digital image of the field of interest was taken every 15 minutes for 24 hours, generating 85 frames that were arranged into sequences in .avi format (Zeiss Axiovert software). Two fields were selected in each well. The nucleus of each cell was followed using manual tracking from the first to the last frame and results recorded (Zeiss LSM Image Browser version 3.2.0.70).

We used mean speed (MS) and final relative distance to the origin (FRDO) as indicators to characterize cell trajectory and motility. Mean cell speed corresponds to the total distance covered during the experiment, divided by the duration of the experiment, which was considered to be representative of cell motility [17]. To assess the distance the cell migrated since its origin to the end of the observation, we analyzed the linear distance between the initial and final cell position that allows the identification of the statistical trend of cells that randomly explore a large area.

### Statistical analysis

Results are presented as mean ± S.E.M. Adequate adjustment of results per gram of adipose tissue were performed when comparing between the fractions and depots of adipose tissue. Normality was assessed by Kolmogorov-Smirnov test. Data for adipose tissue gelatinase activity, prostate cancer cell count and motility (final relative distance to origin), were log_10_-transformed to become normally distributed, whether adjusted or not to adipose tissue weight. One-way ANOVA with between groups' post-hoc Scheffe test or post-hoc Dunnett test, and the independent samples *t*-test, were used as appropriate. Whenever means for different groups wanted to be compared and normality conditions were not satisfied we used the Kruskal-Wallis test followed by Mann Whitney test once a significant P was obtained or only Mann Whitney test.

Statistical analyses were performed with SPSS 17.0. Significance was accepted at P less than 0.05. Details of the statistical analyses were included in each figure legend.

## Results

Some clinicopathological variables, including the body mass index (mean, 26.5 and 95% CI, 24.6-28.5 Kg/m^2^), age at diagnosis (mean, 63.9 and 95% CI, 60.1-67.7 years of age) and prostate specific antigen at diagnosis (mean, 8.2 and 95% CI, 5.3-11.2 ng/dL) presented low dispersion of values between subjects. In order to investigate the proteolytic profile of PP adipose tissue, we evaluated gelatinase activity in conditioned medium from culture of PP adipose tissue explants, according to age at diagnosis, body mass index (BMI), pathologic status and Gleason grade of donors (Table 1). MMP9 was significantly elevated in obese/overweight compared to normoponderal subjects (P = 0.036).

**Table 1:** Gelatinase activity in conditioned medium from primary cultures of periprostatic (PP) adipose tissue explants, according to clinical and pathological characteristics

		MMPs activity in supernatant of PP adipose tissue explant cultures (A.U.)			
	**Demographics**	**MMP2**		**MMP9**	

	**n (%)**	**mean ± S.E.M.**	**P**	**mean ± S.E.M.**	**P**

Age at diagnosis, yrs^a^					
< median (65.1)	13 (52.0)	982.9 ± 154.8	0.591	498.9 ± 71.6	0.624
≥ median (65.1)	12 (48.0)	878.7 ± 111.2		558.3 ± 93.6	

BMI, Kg/m^2 b^					
< 25	11 (44.0)	895.4 ± 135.3	0.739	392.1 ± 48.3	0.036
≥ 25	14 (56.0)	960.3 ± 134.4		635.8 ± 87.5	

Pathologic status^b^					
BPH	5 (20.0)	958.6 ± 97.0	0.795	715.5 ± 142.6	0.242
PCa (< pT3)	14 (56.0)	873.8 ± 150.2		461.9 ± 68.1	
PCa (≥pT3)	6 (24.0)	1026.2 ± 169.8		511.0 ± 128.0	

Gleason grade^a^					
< 7	8 (40.0)	930.7 ± 189.5	0.967	477.0 ± 94.9	0.987
≥ 7	12 (60.0)	920.7 ± 148.6		479.1 ± 81.7	

Results from zymograms performed in supernatants of *in vitro *culture of PP adipose tissue explants (n = 25). ^a ^Independent samples *t*-test or ^b ^one-way ANOVA; A.U., arbitrary units; S.E.M., standard error of mean. MMP2, matrix metalloproteinase 2; MMP9, matrix metalloproteinase 9. BMI, body mass index. BPH, nodular prostatic hyperplasia; PCa, prostate cancer.

**Figure 1:**
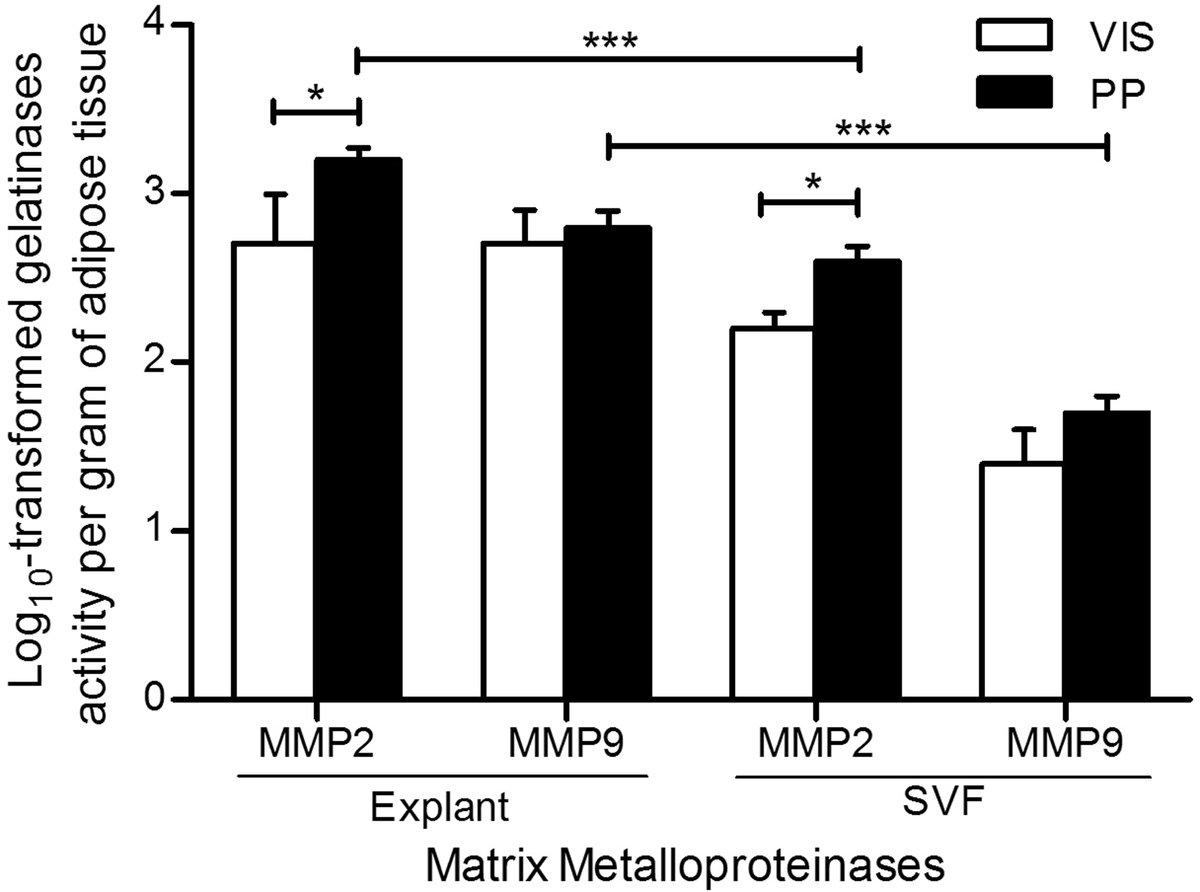
**Gelatinolytic activity of periprostatic (PP) adipose tissue and comparison with visceral pre-peritoneal fat depot**. Analyses were performed in explants and stromal-vascular fraction primary culture of 21 samples of PP adipose tissue and 10 samples of VIS adipose tissue. Independent samples *t*-test was used. *** P < 0.0001 between explants and SVF fraction; * P < 0.05 in the comparison among fat depots. MMP, matrix metalloproteinase; VIS, visceral; PP, periprostatic; SVF, stromal-vascular fraction.

**Figure 2:**
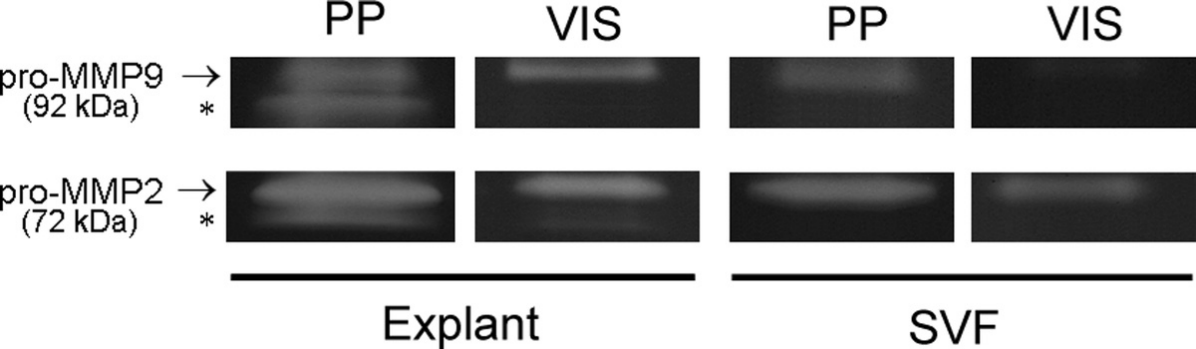
**MMP2 and MMP9 enzymatic activities in supernatants of whole adipose tissue and SVF fraction from VIS and PP depots**. Representative bands corresponding to specific MMP2 and MMP9 are shown. Asterisks indicate active forms of MMP2 and MMP9 while arrows indicate the respective proforms. SVF, stromal-vascular fraction; PP, periprostatic; VIS, visceral; MMP, matrix metalloproteinase.

To understand which fraction of PP adipose tissue contributes to enhanced gelatinase activity, we analyzed paired explant and stromal-vascular fraction cultures from PP adipose tissue (Figure 1). Our results indicate that the proteolytic activity of both MMP2 and MMP9 is higher in cultures of adipose tissue explants than in the correspondent stromal-vascular fractions. A similar proteolytic pattern is present between explants and stromal-vascular fractions of VIS adipose tissue. Additionally, we observed that PP adipose tissues present higher MMP2 but not MMP9 activity, as compared with adipose tissue from a distinct anatomical fat depot (median pre-peritoneal visceral region) (Figure 1). Figure 2 depicts a representative image of zymogram findings.

**Figure 3:**
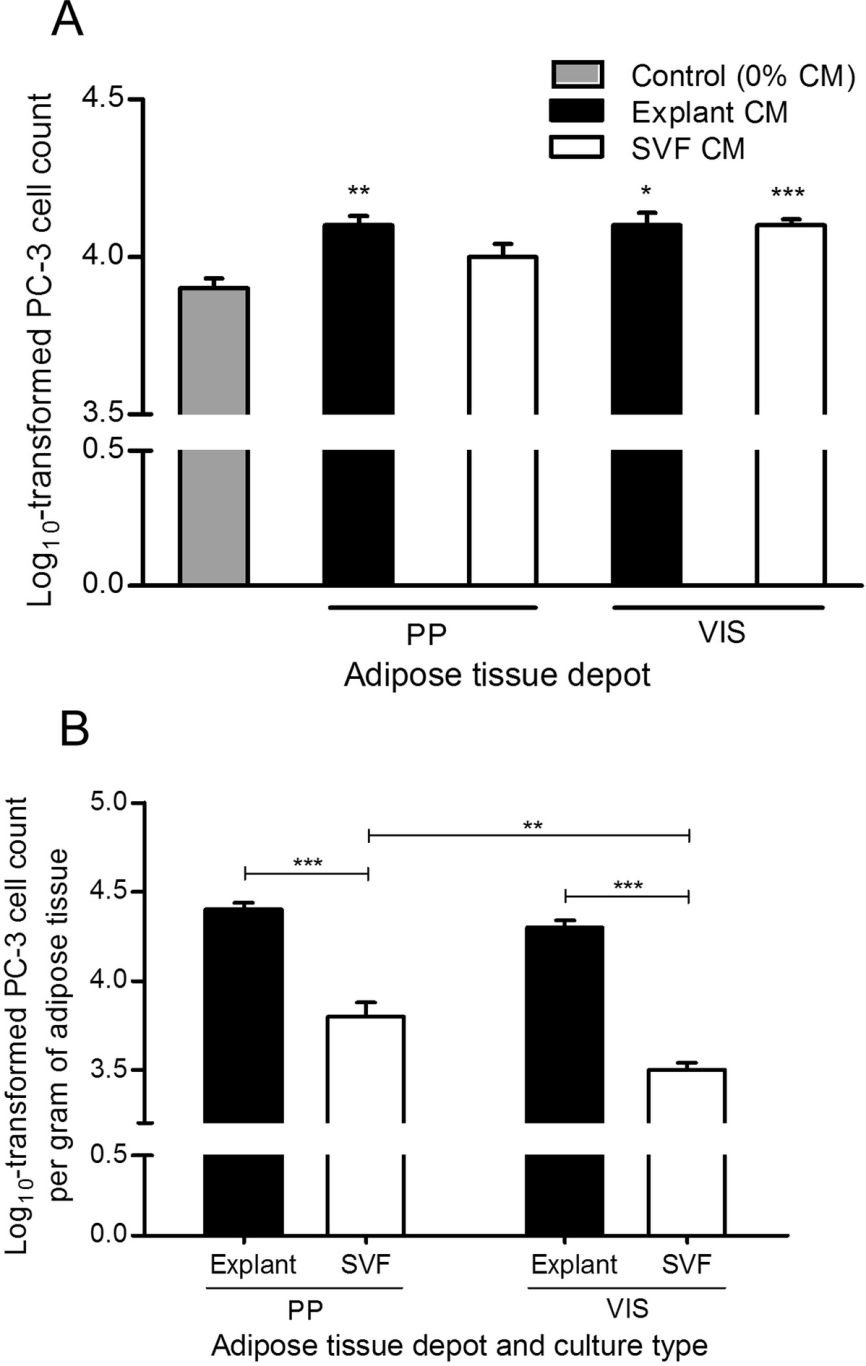
**Influence of conditioned medium from distinct adipose tissue origins in the proliferation of PC-3 cells**. Analyses were performed using conditioned medium of 21 samples of periprostatic (PP) and 10 samples of visceral (VIS) adipose tissue, after explants and stromal-vascular fraction primary cultures. A. Effect of adipose tissue-derived CM on PC-3 cell proliferation, in comparison with control (0% CM) (**P < 0.01 in relation with 0% CM, one-way ANOVA with two-sided post-hoc Dunnett test). B. PC-3 cell proliferation was normalized per gram of adipose tissue and compared according to fat depot and adipose tissue fraction (**P < 0.01 and *** P < 0.0001 between groups, independent samples *t*-test). CM, conditioned medium; PP, periprostatic; SVF, stromal-vascular fraction; VIS, visceral.

Next, to examine whether soluble factors secreted by PP adipose tissue alter tumor cell behavior, its proliferative potential on an aggressive hormone-refractory prostate cancer cell line was investigated. We observed that factors secreted from explants of both PP and VIS adipose tissue increase proliferation of hormone-refractory prostate cancer cells, whereas only VIS SVF culture-derived factors stimulated proliferation (Figure 3A). The log_10_-transformed PC-3 cell count per gram of adipose tissue, was significantly higher after stimulation with explants culture-derived CM compared with SVF, independently of the adipose tissue depot (P < 0.0001) (Figure 3B). Interestingly, the SVF-derived CM of PP adipose tissue had a stronger proliferative effect than SVFs of VIS origin (P = 0.007) (Figure 3B).

**Figure 4:**
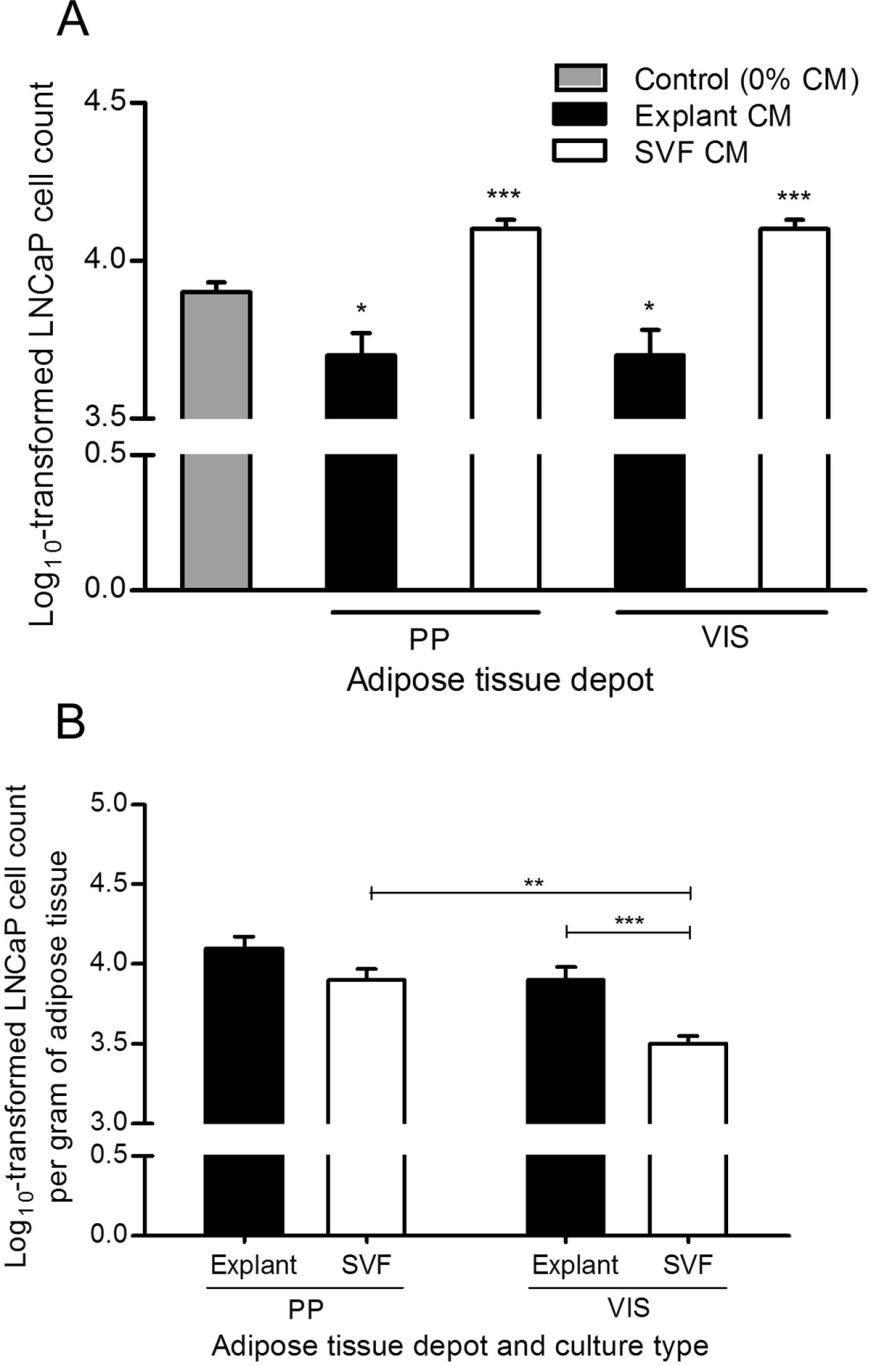
**Influence of conditioned medium from adipose tissue in the proliferation of LNCaP cells**. Analyses were conducted using conditioned medium of periprostatic (PP) and visceral (VIS) adipose tissue from 10 subjects after explants and stromal-vascular fraction primary cultures. A. Influence of adipose tissue-derived CM in LNCaP cell proliferation, in comparison with control (0% CM) (* P < 0.05 and ** P < 0.01, relative to control, two-sided post-hoc Dunnett test). B. Comparison of the effect of CM from distinct adipose tissue depot and fractions in LNCaP proliferation after tissue weight normalization (** P < 0.01 and *** P < 0.0001 between groups, independent samples *t*-test). CM, conditioned medium. SVF, stromal-vascular fraction. PP, periprostatic; VIS, visceral.

The influence of PP adipose tissue secreted factors for cell proliferation of another less aggressive hormone-sensitive prostate cancer cell line was subsequently examined. Interestingly, while these cells also respond to the proliferative stimulus of CM from SVF fraction (P < 0.0001), an inhibitory effect in LNCaP cells was observed with explants CM (P < 0.05), independently of fat depot (Figure 4A). Comparisons between adipose tissue fractions, explants *vs *SVF-derived CM, in LNCaP cell proliferation were conducted using the logarithmically-transformed cell count per gram of adipose tissue (Figure 4B). For VIS but not PP adipose tissue, there was an increased influence of explants compared to SVF CM in LNCaP cell proliferation (P < 0.0001). Furthermore, when compared with VIS SVF CM, the SVF CM from PP adipose tissue increased LNCaP cell proliferation (Figure 4B).

**Figure 5:**
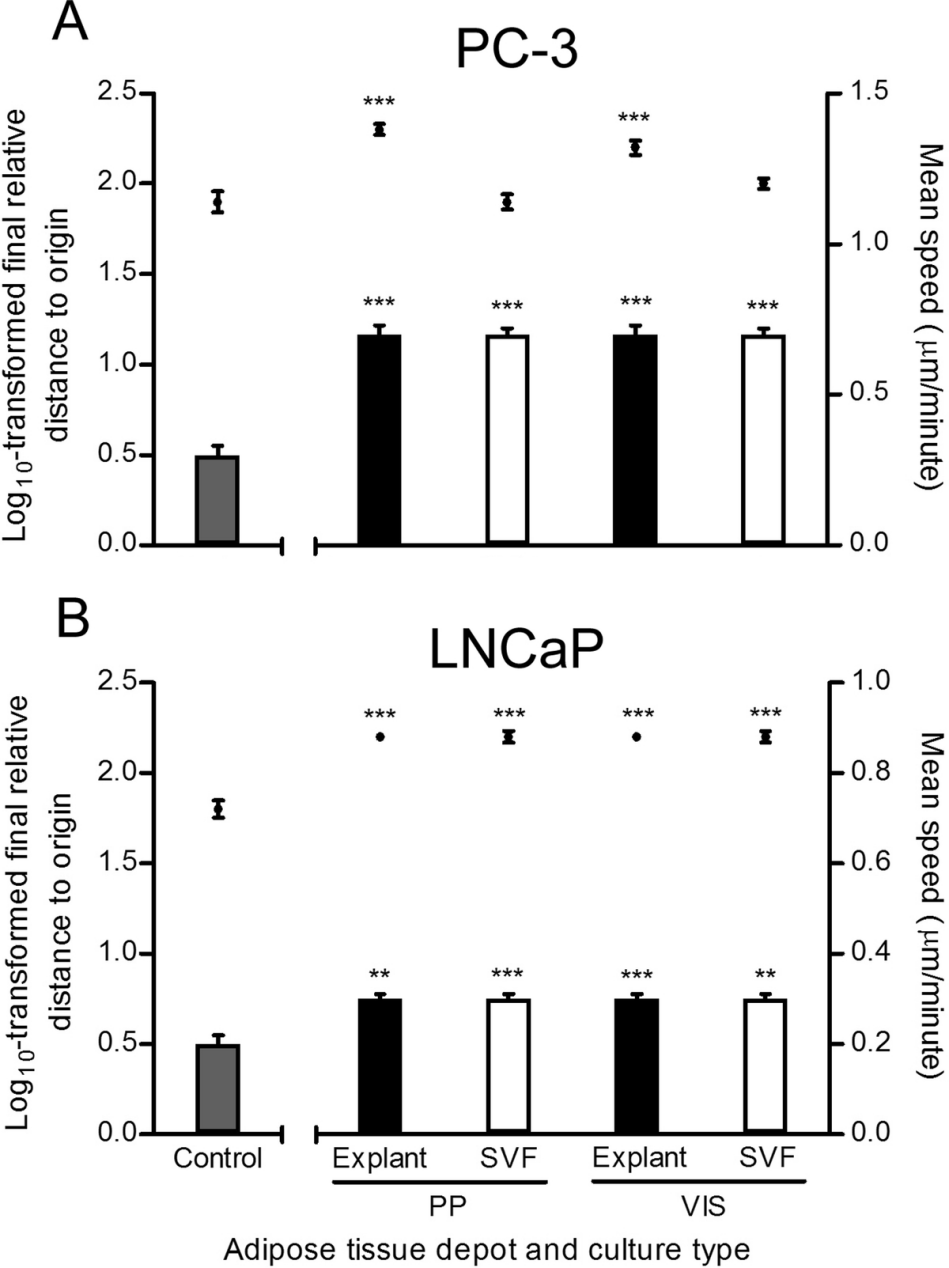
**Motility of PC3 and LNCaP cells upon stimulation of adipose tissue-derived CM from explants and SVF**. Influence of adipose tissue fractions in cell motility parameters. Data represent mean ± SE of at least 20 representative cell trajectories per each tested condition, with conditioned medium of primary adipose tissue cultures from four distinct subjects. Bars represent mean speed (MS) and plots the logarithmically transformed final relative distance to origin (FRDO). A. FRDO and MS of PC-3 cells (*** P < 0.0001 relative to control). B. FRDO and MS of LNCaP cells (** P < 0.01 and *** P < 0.0001 relative to control). In the log-transformed FRDO we used one-way ANOVA with post-hoc Dunnett test (two-sided), whereas the mean speed was analyzed using Kruskal Wallis followed by Mann Whitney test. SVF, stromal-vascular fraction; PP, periprostatic; VIS, visceral.

The enhanced proteolytic activity of PP and VIS adipose tissues led us to investigate their putative effect on prostate cancer cell motility. Therefore, the motile behavior of the PC-3 hormone-refractory and of the LNCaP hormone-sensitive prostate cancer cell lines were analyzed using adipose tissue samples from 4 additional subjects. In the first subject by subject analysis we observed that CM from any adipose tissue fraction or depot elicited, in comparison to untreated cells (control) increased motility, independently of donnor's clinicopathological characteristics (data not shown). Figure 5 shows motile parameters of prostate cancer cells in response to adipose tissue CM. Comparing with control, LNCaP cells stimulated with CM from any fraction or depot always resulted in higher mean speed and final relative distance to origin (FRDO) (Figure 5A). In PC-3 cells, while mean speed was higher for any CM condition compared with control, the FRDO was only increased after stimulation with CM from explants, both from PP and VIS depot (Figure 5B).

**Figure 6:**
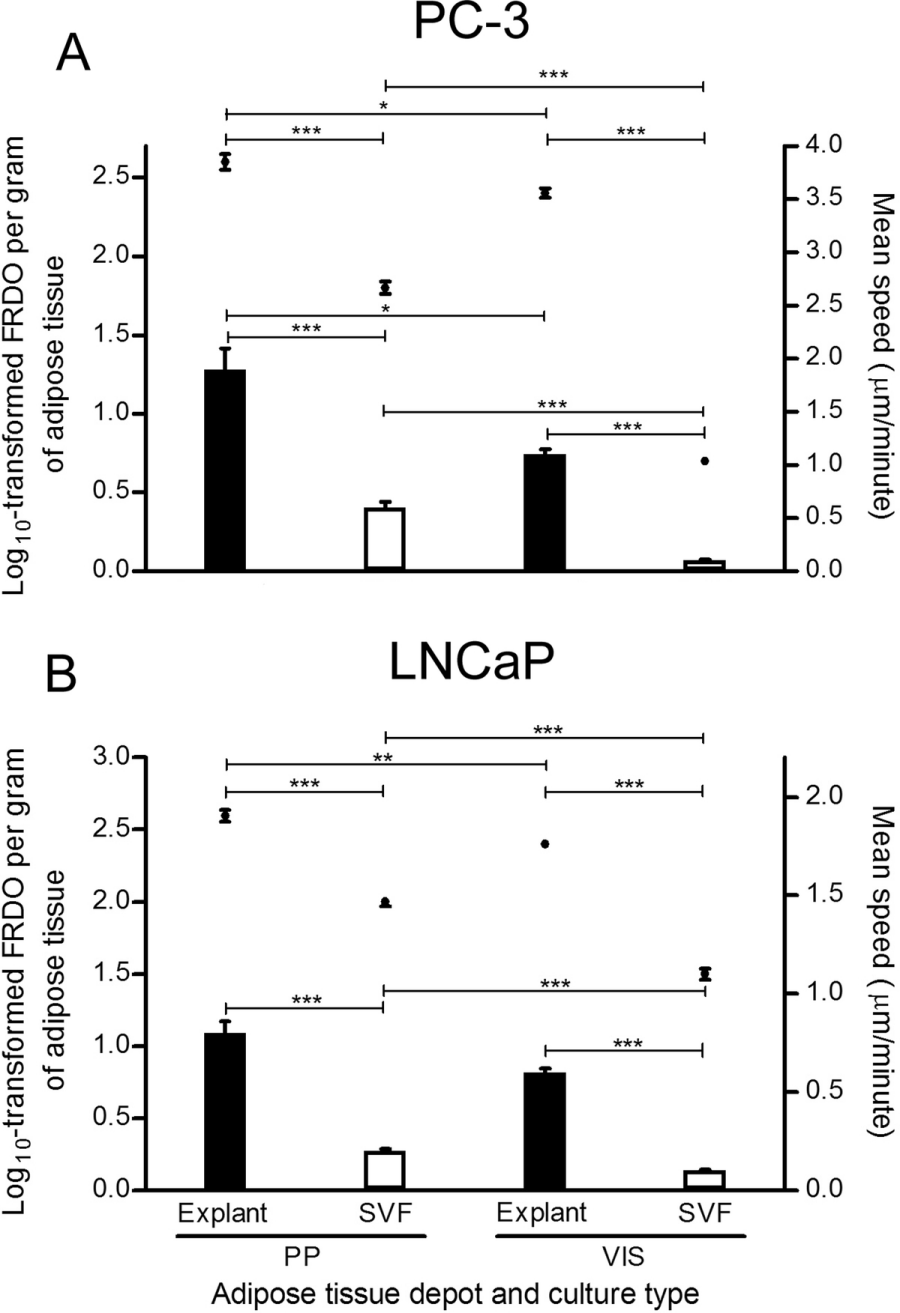
**Motility of PC-3 and LNCaP cells upon stimulation of adipose tissue-derived CM from explants and SVF**. Data represent mean ± SE of at least 20 representative cell trajectories per each tested condition, from four distinct subjects. Bars represent mean speed (MS) per gram of adipose tissue and plots the logarithmically transformed final relative distance to origin per gram of adipose tissue (FRDO). A. FRDO and MS of PC-3 cells (* P < 0.05 and *** P < 0.0001 between treatment conditions). B. FRDO and MS of LNCaP cells (** P < 0.01 and *** P < 0.0001 between conditions). Analyses on MS were performed with Mann Whitney test, whereas FRDO was analyzed using independent samples *t*-test.SVF, stromal-vascular fraction; PP, periprostatic; VIS, visceral.

**Figure 7:**
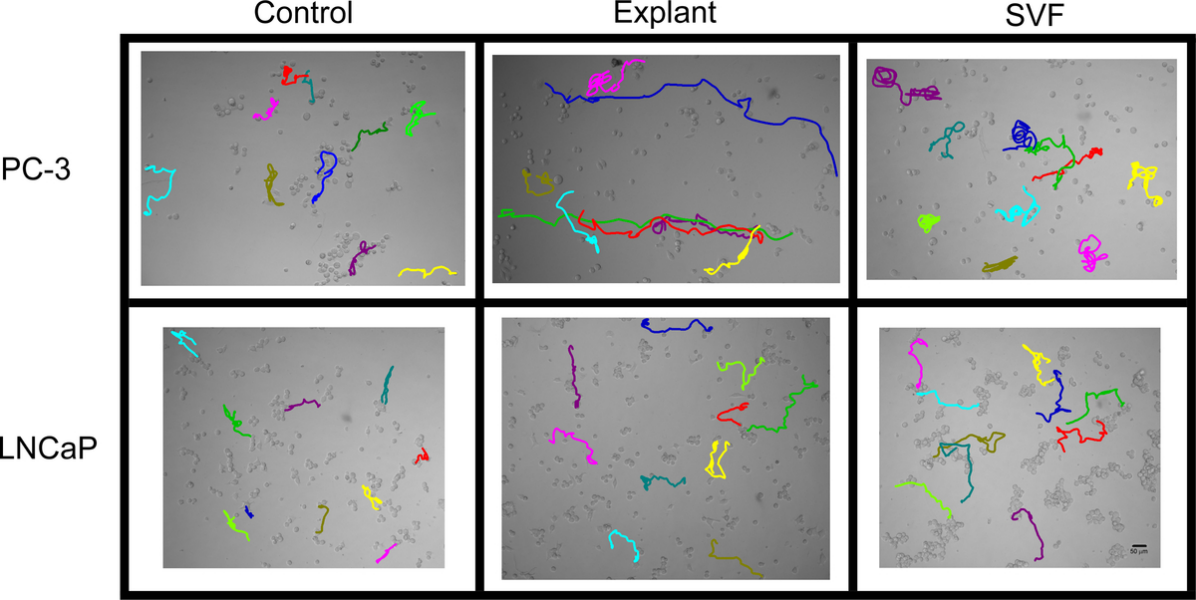
**Representative example of cell tracking and cancer cell trajectories after stimulation with periprostatic adipose tissue-derived CM**. Sequential displacements of cells were captured by manual cell tracking and are represented as color lines. SVF, stromal-vascular fraction.

After adjustment of motility parameters to adipose tissue weight, in order to compare different culture types and depots, only the LNCaP cells mean speed was not statistically different between PP and VIS depot. Otherwise, motile parameters were higher after stimulation with CM from PP depot (Figure 6). For both PC-3 (Figure 6A) and LNCaP (Figure 6B) cells stimulated with explant-derived CM from PP and VIS adipose tissue, the mean speed and FRDO were significantly higher in comparison to SVF (P < 0.0001). Figure 7 shows a representative example of cell tracking in both cancer cell lines, using CM from PP adipose tissue.

## Discussion

Prostate cancers frequently have a indolent course even if left without active treatment [18]. However, clinically relevant disease with significant morbidity and mortality also occurs in a significant number of patients [19]. The mechanisms responsible for this aggressive behavior remain elusive, albeit it is well established that the supporting tumor microenvironment has a decisive role in controlling prostate cancer growth, invasion and metastasis [20].

Cancer-implicated mammary and colonic fat pads [11,21] are physically close to epithelial cells, whereas in prostate there is initially a capsular-like structure separating the PP fat from tumor cells. Nevertheless, frequently prostate tumors infiltrate the PP fat pad by transposing or infiltrating the physical barriers, resulting in immediate proximity to adipose tissue. Once extension beyond the capsule occurs, the PP adipose tissue-secreted factors, extracellular matrix components or direct cell-cell contact may influence the phenotypic behavior of malignant cells. Recent studies observed that PP adipose tissue thickness was linked to prostate cancer severity [8], while its secretory profile associated with advanced disease [7]. In the present study, we found that PP adipose tissue-derived conditioned media may potentiate prostate cancer aggressiveness through modulation of metalloproteinases activity, and by promoting cancer cell proliferation and migration.

In tumors, cancer cells are not the only source of MMPs. In our study, MMP9 activity was significantly elevated in the PP adipose tissue of overweight/obese men (BMI ≥ 25 Kg/m^2^), implying excess body fat and the PP fat depot in the modulation of extra-capsular cancer cells' microenvironment. Concordantly, other studies found MMP9 to be positively correlated with BMI [22]. Further research is warranted to uncover the effects of MMPs in association with distinct obesity grades. In our sample only two subjects presented BMI > 30 Kg/m^2^, limitating such approach.

Matrix metalloproteinases are proteolytic enzymes that regulate many cell mechanisms with prominence in cancer biology [23]. Their expression in prostate tumors is related with disease progression and metastasis [24], whereas MMP9 was shown to increase growth factors bioavailability and to elicit epithelial-to-mesenchymal transition in tumor cells [25,26], therefore promoting an aggressive phenotype. A recent report indicated that oesophageal tumors from obese patients express more MMP9 and that co-culture of VIS adipose tissue explants with tumor cells up-regulated MMP2 and MMP9 [27]. Remains undetermined the influence of PP adipose tissue in the expression of MMPs by prostate cancer cells, which might further contribute towards an aggressive phenotype. Noteworthy, cancer-derived factors stimulate other surrounding cells, including adipose tissue cells, to synthesize MMPs [15].

In an effort to understand if the effects of PP adipose tissue extend to other aggressiveness characteristics, we used adipose tissue-derived CM to perform cell proliferation assays in prostate cancer cell lines. We found that CM from *in vitro *culture of adipose tissue explants stimulated the proliferation of hormone-refractory prostate cancer cells. Conversely, this media inhibited growth in hormone-sensitive cells.

It is well-established that adipose tissue secretes a wide array of molecules [28]. These adipokines, exclusively or partially secreted by adipocytes or stromal-vascular fraction cells, are likely to have a role in modulating the risk of cancer progression [1,29,30]. Few studies examined the effect of adipocytes in prostate cancer cells growth [12,13]. While a proliferative effect was observed in hormone-refractory PC-3 cells, these findings didn't replicate in LNCaP cells [13]. In fact, the mitogenic and anti-apoptoptic effects of several adipokines, alone and combined, in prostate cancer cell growth (e.g. leptin, IL-6, insulin-like growth factor 1, IGF-1), seems to be limited to hormone-refractory prostate cancer cells [12,31-34]. Previous studies also report on the suppression of LNCaP cell growth as response to adipokines (e.g. TNF-α, decreased expression of vascular endothelial growth factor, VEGF), not observed in hormone-refractory cells [13,35-37].

Contrary to explants, CM from SVF cultures induces cancer cell proliferation, independently of cell line, except for the SVF from PP adipose tissue in PC-3 cells. Cells that constitute the SVF fraction of adipose tissue, where macrophages have a modulatory role, are known to secrete several angiogenic and antiapoptotic factors [38-40], which ultimately can impact prostate cancer cells growth. The lack of proliferative effect observed for the SVF fraction from PP adipose tissue may partially be due to the reported low number of macrophages in PP fat depot [7], diminishing the proliferative stimulus in prostate cancer cells.

Progression to an invasive and metastatic phenotype is responsible by prostate cancer mortality and morbidity. The increased cellular motility is another parameter associated with increased metastatic potential [41,42]. By employing time-lapsed imaging, we found that factors produced by whole adipose tissue cultures (explants) increased significantly the migration speed and the final relative distance to origin of both PC-3 and LNCaP cells compared with control. Only the SVF fraction-derived CM effect in the final relative distance to origin of PC-3 cells, was not increased compared with control.

The mechanisms involved in tumor cell movement are far from fully elucidated, although various biophysical processes are considered to be involved [41]: in order for a cell to move it must be polarized or have a sense of directionality; polarity is accompanied by 1) lamellipodia protrusion at the leading edge, followed by 2) detachment of the cell's rear end and subsequent 3) transcellular contractility. These mechanisms are modulated by the activation of several signaling pathways, such as PI3K, ERK/MAPK and c-Src tyrosine kinase [41], which are known downstream signals of adipokines [43]. In fact, many adipokines (e.g. IGF-1, osteopontin, leptin, adiponectin, VEGF, thrombospondin, interleukin-8 and IL-6) have been shown to modulate different steps of cell motile behavior [44-56]. The repetitive and coordinated cycling of these processes results in productive locomotion of the cell. Several key pathways and molecules involved in this process can be induced by factors secreted by adipose tissue, hence supporting the increased motility we found in stimulated prostate cancer cells. Nevertheless, besides the influence of extrinsic factors, migratory tumor cells also present autocrine growth factor signaling systems [57]. We disclose any potential bias from inadvertent selection using manual cell tracking analysis, urging careful interpretation of motility findings. Further studies using migration assays to extend and confirm our results are warranted.

Adipose tissue is a heterogeneous organ that consists of multiple cell types: adipocyte fraction, which contains lipid-loaded adipocytes, and stromal-vascular fraction, which includes preadipocytes, endothelial cells, fibroblasts, stem cells, macrophages and other immune cells [58]. The fractions of adipose tissue differ in that while explants reflect an organotypic cell culture system of whole adipose tissue, the major characteristic of stromal-vascular fraction culture is the depletion of adipocytes and absence of extracellular matrix. In order to investigate which fraction influenced tumor cells, we cultured paired explants and stromal-vascular fraction cells. To allow comparison between depots and adipose tissue fractions, the cell count was adjusted per gram of adipose tissue. Interestingly, our findings showed that media from explants and PP adipose tissue depot presented the higher gelatinolytic activity per gram of adipose tissue, compared with SVF cultures- and VIS adipose tissue-derived media. Although the amount of MMP9 has been described to be higher in stromal-vascular fraction of adipose tissue compared with adipocytes [22], the latter have greater plasticity to increase MMPs expression when interacting with other cells in adipose tissue [22,59]. The increased activity of metalloproteinases in CM from adipose tissue explants in culture compared with SVF, likely reflect the additive effect or interaction between cells of the stromal-vascular fraction plus adipocytes. We found that MMP2 activity was increased in PP versus VIS adipose tissue supernatants. Although there is no evidence of MMP2 role in adipose tissue/cancer cells crosstalk, recent findings suggest MMP2 is up-regulated in tumor cells co-cultured with adipose tissue explants and that its expression and activation is modulated by several adipokines (e.g. Wdnm1-like and visfatin) [27,60,61]. Additionally, other MMPs, notably MMP11, have been shown to be correlated with breast cancer-induced adipocyte's activated state [11,62]. If confirmed, our findings may reveal a novel specific proteinase expression and activity pattern in PP adipose tissue favorable to prostate cancer progression.

In this study, proliferation was increased with CM from PP and VIS explants versus SVF CM in PC-3 cells, whereas LNCaP cells only proliferated significantly more with VIS explants compared to VIS SVF. As the highest proliferation was seen following stimulation with CM from explants we speculate adipocytes may be the main effectors. Other studies also found a proliferative effect of adipocytes in prostate cancer cells [12,13]. Adipocytes add significantly to the proliferative effect in hormone-refractory prostate cancer cells, even though the adipokines responsible by these results have yet to be determined. Alternatively, since explants culture preserve the paracrine signals by maintaining the existing crosstalk among the different cell types [63], we hypothesize that the higher proliferative stimulus conferred by explants CM likely reflects a co-stimulatory and/or additive effect of adipokines produced by adipocytes and by the stromal vascular fraction cells.

Explants-derived CM, whether from VIS or PP origin exerted consistently, also across cell lines, an increased effect in migration speed and final relative distance to origin, when compared with SVF fraction. It is possible that explants CM, which reveal the secretory profile of adipocytes plus stromal-vascular cells, produce more motile factors and exclusive secretion of others (e.g. leptin and adiponectin), thereby resulting in increased total distance/mean speed and final relative distance to origin of prostate cancer cells.

The anatomical origin of adipose tissue accounts for increased gelatinolytic activity and different proliferative and migratory stimulus. CM from PP results in higher log_10_-transformed PC-3 and LNCaP cell count per gram of adipose tissue, only when SVF CM was used. Furthermore, adipose tissue from PP origin exerted the stronger motile effect (of both analyzed parameters) in PC-3 cells compared to VIS depot, independently of the culture type. In LNCaP cells only the PP explants-derived CM didn't impact the mean speed more than CM from VIS explants. These findings suggest that VIS and PP fat pads may have distinct relative cellular composition or are differently programmed to secrete molecules involved in the regulation of cell proliferation and motility. We recently found increased amount of adipose stem cells (CD34^+^/CD45^-^/CD31^-^/CD146^-^) in PP compared with VIS adipose tissue (Ribeiro R, unpublished observations).

Tumor cell progression depends on itself as well as on the surrounding microenvironment, which is able to influence proliferation, migration and metastatic behavior of tumor cells by modulating the extracellular matrix and growth factor production [64]. If the tissues where tumor cells exist provide the missing extrinsic signals, then cells will proliferate and acquire an invasive phenotype, which may lead to metastasis. Whole periprostatic fat, not only stromal vascular fraction cells, seems to warrant the necessary factors to induce a specific microenvironment for prostate cancer tumor cells, which ultimately may result, as we found, in tumor cell survival, increased motility and availability of extracellular proteases. During cell migration, pericellular proteolysis of extracellular matrix is important for cell protrusion.

The increased production of MMPs found in PP adipose tissue can fuel invasive and metastatic behavior of PP fat-infiltrating prostate cancer cells.

## Conclusions

In this study we found that PP adipose tissue-derived factors may potentiate prostate cancer aggressiveness through modulation of metalloproteinases activity, and by promoting cancer cell proliferation and motility. In addition, results indicate that factors secreted by whole periprostatic fat induce a favorable microenvironment for hormone-refractory prostate cancer tumor cells. These previously unrecognized findings suggest a role for PP adipose tissue in prostate cancer progression, and as a candidate explanatory mechanism to the causally invoked association between obesity and aggressive prostate cancer.

## Abbreviations

BMI: Body mass index; BPH: Nodular prostatic hyperplasia; CM: Conditioned medium; FRDO: Final relative distance to origin; IL-6: Interleukin 6; LNCaP: Hormone-sensitive prostate cancer cell line; MMP: Matrix metalloproteinase; MS: Mean speed; PC-3: Hormone-refractory prostate cancer cell line; PP: Periprostatic; SVF: Stromal-vascular fraction; VIS: Visceral.

## Competing interests

The authors declare that they have no competing interests.

## Authors' contributions

RR, VC and CM performed most of the experiments. MJO performed the zymography, assisted with the cell tracking experiment and edited the manuscript. MF assisted with some of the *in vitro *experiments and edited the manuscript. AF, PP, CL, FL, AM, VS, JSM, JO and FP collected adipose tissue and clinicopathologic patient information and edited the manuscript. RR and RM performed the statistical analysis. RR, CM, AMP, CL and RM designed the experiments and edited the manuscript. RR wrote the manuscript. All authors read and approved the final manuscript.
